# Complete genomic sequences of *Pseudooceanicola* and *Leisingera* species isolated from surface seawater: potential polyolefin degraders and bioplastic producers

**DOI:** 10.1128/mra.00584-25

**Published:** 2025-07-31

**Authors:** Ryo Iizuka, Sotaro Uemura

**Affiliations:** 1 Department of Biological Sciences, Graduate School of Science, The University of Tokyo 13143, Bunkyo-ku, Tokyo, Japan; 2 Core Research for Evolutional Science and Technology (CREST), Japan Science and Technology Agency 13501, Kawaguchi, Saitama, Japan; Indiana University, Bloomington, Bloomington, Indiana, USA

**Keywords:** polyolefin, bioplastic, *Pseudooceanicola*, *Leisingera*

## Abstract

Two bacterial strains, a novel *Pseudooceanicola antarcticus* strain and a new *Leisingera* species, were isolated from surface seawater through an enrichment culture using a lower-molecular-weight polyolefin. Their complete genomes contain genes for alkane degradation and polyhydroxyalkanoate synthesis, indicating their potential for converting polyolefins into biodegradable plastics.

## ANNOUNCEMENT

Plastic pollution in marine environments represents a growing global challenge (1–4). We present the isolation and genome sequencing of two marine bacterial strains with potential polyolefin-degrading abilities.

Surface seawater was collected at Shirarahama Beach, Wakayama, Japan (33°40′53.0″N 135°20′39.0″E) on August 15, 2024. The seawater was incubated with 1% LUCANT HC-40 (Mitsui Chemicals), a lower-molecular-weight polyolefin consisting of ethylene-α-olefin co-oligomers. After 23 days of incubation at 25°C, a portion of LUCANT substrate was directly streaked onto agar plates using an inoculation loop and incubated for 4 days. Strains S2H3 and S2M4 were isolated from plates containing artificial seawater medium (5–7) and Marine Broth 2216 (Difco), respectively. Subsequent 16S rRNA gene analysis (6, 7) identified them as members of the *Pseudooceanicola* and *Leisingera* genera, respectively. The strains were cultured aerobically at 25°C for genomic sequencing: S2H3 in artificial seawater medium for 18 h and S2M4 in Marine Broth for 5 days.

Genomic DNA was extracted using Genomic-tip 20/G (Qiagen) and further purified with Short Read Eliminator (PacBio) and 1.8× DNA Clean Beads (MGI) for S2H3, or with 0.94× ProNex Beads (Promega) for S2M4. For PacBio library preparation, genomic DNA was sheared into 10–25 kbp fragments using Megaruptor 3 (Diagenode). No size selection was performed. Libraries prepared using SMRTbell prep kit 3.0 and SMRTbell gDNA amplification kit were treated with Revio Polymerase Kit (all from PacBio). Sequencing was performed using the Revio system (PacBio). SMRT Link (v13.1.0.221970) (PacBio) was used to trim adapter sequences from the sequencing reads. Circular consensus sequences with average base quality values below 20 were removed to obtain HiFi reads. These reads were filtered using lima (v2.12.0) (https://github.com/pacificbiosciences/barcoding) and pbmarkdup (v1.0.3) (https://github.com/PacificBiosciences/pbmarkdup) to remove the ultra-low PCR adapters and PCR duplicates, respectively. Filtlong (v0.2.1) (https://github.com/rrwick/Filtlong) was used to exclude short HiFi reads (<1,000 bp). The remaining reads were assembled using Flye (v2.9.3-b1797), which automatically trimmed overlaps to produce circular contigs (8). DFAST (v1.6.0) was used for taxonomic classification, average nucleotide identity (ANI) calculation, and annotation (9–11). Default settings were used for all software.

The S2H3 genome was assembled into two circular contigs, corresponding to a chromosome and a plasmid (Table 1). Phylogenomic analysis showed that its closest relative is *Pseudooceanicola antarcticus* CGMCC 1.12662^T^ (GCF_900215355.1), with ANI of 98.19%, indicating a novel strain of *P. antarcticus* (12, 13). To our knowledge, this report is the first presentation of the complete genomic sequence of *P. antarcticus*. The S2M4 genome included a chromosome and three secondary replicons (Table 1). It was closely related to that of *Leisingera caerulea* DSM 24564^T^ (GCF_000473325.1) (14, 15), with ANI of 84.24%, representing a new species within the *Leisingera* genus. Both genomes encode enzymes involved in the aerobic oxidation of alkanes, including alkane 1-monooxygenase, which likely contribute to polyolefin degradation (16–18). They also encode class I polyhydroxyalkanoate synthase, suggesting the capability for converting polyolefin degradation products into biodegradable plastics (19, 20). This genomic information provides insights into the mechanisms by which marine bacteria might convert synthetic plastics into biodegradable alternatives.

**TABLE 1:** Sequencing statistics and genomic features of strains S2H3 and S2M4

	S2H3^*e*^	S2M4
BioSample accession number	SAMD00896922	SAMD00896923
Sequencing results		
Number of reads	32,034	39,600
Sum of length (bp)	248,239,687	249,148,960
Read N50 (bp)	9,193	7,137
SRA accession no.	DRR658062	DRR658063
Assembly results		
Length (bp)		
Chromosome	4,257,145	3,389,420
Secondary replicon 1	36,462^*a*^	574,606^*b*^
Secondary replicon 2	–	250,224^*c*^
Secondary replicon 3	–	115,470^*d*^
GC content (%)		
Chromosome	66.4	63.9
Secondary replicon 1	62.5	62.3
Secondary replicon 2	–	63.1
Secondary replicon 3	–	65.1
Coverage (×)	57.8	57.5
Number of protein-coding sequences		
Chromosome	4,039	3,218
Secondary replicon 1	33	523
Secondary replicon 2	–	211
Secondary replicon 3	–	79
Number of rRNA genes		
Chromosome	9	12
Secondary replicon 1	0	3
Secondary replicon 2	–	2
Secondary replicon 3	–	0
Number of tRNA genes		
Chromosome	53	66
Secondary replicon 1	0	3
Secondary replicon 2	–	4
Secondary replicon 3	–	0
GenBank accession number		
Chromosome	AP040859	AP040861
Secondary replicon 1	AP040860	AP040862
Secondary replicon 2	–	AP040863
Secondary replicon 3	–	AP040864

^*a*^ The circular contig has a small size (~36.5 kb), different GC content (62.5%) compared with the chromosome (66.4%), contains a replication initiator protein gene, and lacks essential chromosomal genes, suggesting that it likely functions as a plasmid.

^*b*^ The circular contig (~575 kb) contains rRNA and tRNA genes, as well as a small number (3.88%) of the single-copy essential marker genes used in the CheckM (11, 21). The contig may function as a chromid.

^*c*^ The circular contig (~250 kb) contains rRNA and tRNA genes but lacks essential chromosomal genes. This contig may act as a chromid or plasmid.

^*d*^ The circular contig (~115 kb) has a size typical of plasmids, contains a replication initiator protein gene, and lacks essential chromosomal genes, suggesting that it likely functions as a plasmid.

^*e*^ “–”, not applicable.

## Data Availability

S2H3 and S2M4 are associated with BioProject accession number PRJDB20523, and the accession numbers are listed in Table 1.
